# (*E*)-*N*′-[1-(4-Hydroxy­phen­yl)ethyl­idene]-2-(quinolin-8-yl­oxy)acetohydrazide methanol solvate

**DOI:** 10.1107/S1600536809006461

**Published:** 2009-02-28

**Authors:** Jun Tan

**Affiliations:** a College of Biological and Chemical Engineering, Jiaxing University, Jiaxing 314001, People’s Republic of China

## Abstract

In the title compound, C_19_H_17_N_3_O_3_·CH_4_O, the mean planes of the benzene ring and the quinoline rings make a dihedral angle of 75.5 (2)°. The acetohydrazide mol­ecules are connected *via* pairs of inter­molecular O—H⋯O hydrogen bonds into inversion dimers, and the methanol solvent mol­ecule is linked to the acetohydrazide mol­ecule *via* inter­molecular N—H⋯O and bifurcated O—H⋯(N,O) hydrogen bonds.

## Related literature

For background on the coordination chemistry of 8-hydroxy­quinoline and its derivatives, see: Chen & Shi (1998 ▶). For related structures, see: Wen *et al.* (2005 ▶). For reference structural data, see: Allen *et al.* (1987 ▶).
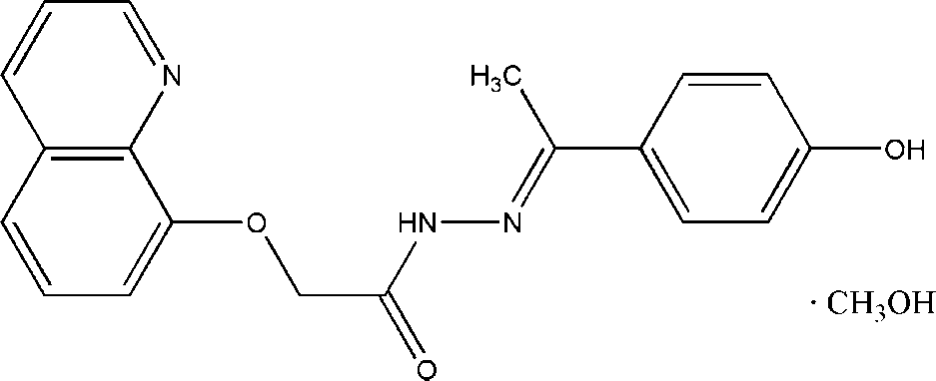


## Experimental

### 

#### Crystal data

C_19_H_17_N_3_O_3_·CH_4_O
*M* *_r_* = 367.40
Triclinic, 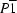

*a* = 9.552 (3) Å
*b* = 10.622 (2) Å
*c* = 10.665 (4) Å
α = 70.055 (5)°
β = 83.033 (4)°
γ = 65.845 (4)°
*V* = 927.9 (5) Å^3^
*Z* = 2
Mo *K*α radiation
μ = 0.09 mm^−1^
*T* = 295 K
0.20 × 0.18 × 0.15 mm

#### Data collection

Bruker SMART CCD diffractometer
Absorption correction: multi-scan (*SADABS*; Sheldrick, 1996 ▶) *T*_min_ = 0.982, *T*_max_ = 0.986
4927 measured reflections
3261 independent reflections
2430 reflections with *I* > 2σ(*I*)
*R*_int_ = 0.021

#### Refinement

*R*[*F* ^2^ > 2σ(*F* ^2^)] = 0.050
*wR*(*F* ^2^) = 0.143
*S* = 1.08
3261 reflections
246 parameters
H-atom parameters constrained
Δρ_max_ = 0.17 e Å^−3^
Δρ_min_ = −0.20 e Å^−3^

### 

Data collection: *SMART* (Siemens, 1996 ▶); cell refinement: *SAINT* (Siemens, 1996 ▶); data reduction: *SAINT*; program(s) used to solve structure: *SHELXS97* (Sheldrick, 2008 ▶); program(s) used to refine structure: *SHELXL97* (Sheldrick, 2008 ▶); molecular graphics: *SHELXTL* (Sheldrick, 2008 ▶); software used to prepare material for publication: *SHELXTL*.

## Supplementary Material

Crystal structure: contains datablocks global, I. DOI: 10.1107/S1600536809006461/hb2916sup1.cif

Structure factors: contains datablocks I. DOI: 10.1107/S1600536809006461/hb2916Isup2.hkl

Additional supplementary materials:  crystallographic information; 3D view; checkCIF report

## Figures and Tables

**Table 1:** Hydrogen-bond geometry (Å, °)

*D*—H⋯*A*	*D*—H	H⋯*A*	*D*⋯*A*	*D*—H⋯*A*
O3—H3⋯O2^i^	0.82	1.85	2.647 (3)	165
O4—H4⋯N1	0.82	1.96	2.773 (3)	174
O4—H4⋯O1	0.82	2.60	3.036 (3)	115
N2—H2⋯O4	0.86	2.10	2.856 (3)	146

Symmetry code: (i) 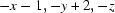.

**Fig. 1.**
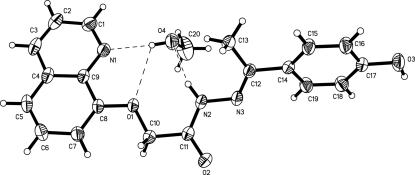
The molecular structure of (I), with displacement ellipsoids drawn at the 30% probability level. The dashed lines indicate hydrogen bonds.

**Fig. 2.**
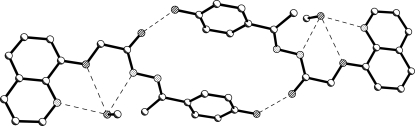
The structure of the dimers formed via hydrogen bonds, H atoms have been omitted for clarity. The dashed lines indicate hydrogen bonds.

## supplementary crystallographic information

### Comment

8-Hydroxyquinoline and its derivatives constitute well known ligands in coordination chemistry (Chen & Shi, 1998). In our search for new extractants of metal ions and biologically active materials, the title compound, (I), has been synthesized. We report here its crystal structure. All bond lengths and angles are normal (Allen *et al.*, 1987), and are comparable to those in the related compound *N'*-(2-Fluorobenzylidene) -2-(quinolin-8-yloxy)-acetohydrazide methanol solvate (Wen *et al.*, 2005). The mean planes of the benzene ring and the quinoline rings make a dihedral angle of 75.5 (2)°. In the crystal structure, the methanol molecule is linked to the C_19_H_17_N_3_O_3_ molecule *via* intermolecular O—H···O, N—H···O and O—H···N hydrogen bonds (Fig. 1), intermolecular O—H···O hydrogen bonds (Table 1) link the molecules into centrosymmetric dimers (Fig. 2).

### Experimental

2-(Quinolin-8-yloxy)acetohydrazide (2.18 g, 10 mmol), 1-(4-hydroxyphenyl)ethanone (1.36 g, 10 mmol), ethanol (40 ml) and some drops of acetic acid were added to a 100 ml flask, and refluxed for 3 h. After cooling to room temperature, the mixture was filtered. Pale yellow blocks of (I) were obtained by slow evaporation of a acetone-methanol (1:2, *v*/*v*) solution over a period of 3 d. Analysis calculated for C_20_H_21_N_3_O_4_: C 65.38, H 5.76, N 11.43%; found: C 65.76, H 5.47, N 11.67%.

### Refinement

All H atoms were initially located in a difference Fourier map. The methylene H atoms were constrained to an ideal geometry, with C—H = 0.93 Å for aryl, 0.97 Å for the methylene, and 0.96 Å for the methyl H atoms, O—H = 0.82 Å and N—H = 0.86 Å. *U*_iso_(H) = 1.2*U*_eq_(C,*N*), or 1.5*U*_eq_(C) for the methyl groups, and 1.5*U*_eq_(O).

### Crystal data

**Table:**

C_19_H_17_N_3_O_3_·CH_4_O	*Z* = 2
*M**_r_* = 367.40	*F*(000) = 388
Triclinic, *P*1	*D*_x_ = 1.315 Mg m^−^^3^
Hall symbol: -P 1	Mo *K*α radiation, λ = 0.71073 Å
*a* = 9.552 (3) Å	Cell parameters from 1866 reflections
*b* = 10.622 (2) Å	θ = 2.5–26.5°
*c* = 10.665 (4) Å	µ = 0.09 mm^−^^1^
α = 70.055 (5)°	*T* = 295 K
β = 83.033 (4)°	Block, light yellow
γ = 65.845 (4)°	0.20 × 0.18 × 0.15 mm
*V* = 927.9 (5) Å^3^	

### Data collection

**Table:**

Bruker SMART CCD diffractometer	3261 independent reflections
Radiation source: fine-focus sealed tube	2430 reflections with *I* > 2σ(*I*)
graphite	*R*_int_ = 0.021
φ and ω scans	θ_max_ = 25.0°, θ_min_ = 2.0°
Absorption correction: multi-scan (*SADABS*; Sheldrick, 1996)	*h* = −11→8
*T*_min_ = 0.982, *T*_max_ = 0.986	*k* = −12→7
4927 measured reflections	*l* = −12→12

### Refinement

**Table:**

Refinement on *F*^2^	Primary atom site location: structure-invariant direct methods
Least-squares matrix: full	Secondary atom site location: difference Fourier map
*R*[*F*^2^ > 2σ(*F*^2^)] = 0.050	Hydrogen site location: inferred from neighbouring sites
*wR*(*F*^2^) = 0.143	H-atom parameters constrained
*S* = 1.08	*w* = 1/[σ^2^(*F*_o_^2^) + (0.0478*P*)^2^ + 0.5262*P*] where *P* = (*F*_o_^2^ + 2*F*_c_^2^)/3
3261 reflections	(Δ/σ)_max_ < 0.001
246 parameters	Δρ_max_ = 0.17 e Å^−^^3^
0 restraints	Δρ_min_ = −0.19 e Å^−^^3^

### Special details

**Table:**

Geometry. All e.s.d.'s (except the e.s.d. in the dihedral angle between two l.s. planes) are estimated using the full covariance matrix. The cell e.s.d.'s are taken into account individually in the estimation of e.s.d.'s in distances, angles and torsion angles; correlations between e.s.d.'s in cell parameters are only used when they are defined by crystal symmetry. An approximate (isotropic) treatment of cell e.s.d.'s is used for estimating e.s.d.'s involving l.s. planes.
Refinement. Refinement of *F*^2^ against ALL reflections. The weighted *R*-factor *wR* and goodness of fit *S* are based on *F*^2^, conventional *R*-factors *R* are based on *F*, with *F* set to zero for negative *F*^2^. The threshold expression of *F*^2^ > σ(*F*^2^) is used only for calculating *R*-factors(gt) *etc*. and is not relevant to the choice of reflections for refinement. *R*-factors based on *F*^2^ are statistically about twice as large as those based on *F*, and *R*- factors based on ALL data will be even larger.

### Fractional atomic coordinates and isotropic or equivalent isotropic displacement parameters (Å^2^)

**Table:**

	*x*	*y*	*z*	*U*_iso_*/*U*_eq_	
O1	0.32959 (17)	0.65533 (18)	0.12916 (16)	0.0512 (4)	
O2	0.0115 (2)	0.8800 (2)	−0.09376 (17)	0.0620 (5)	
O3	−0.83771 (19)	1.1439 (3)	0.2526 (2)	0.0726 (6)	
H3	−0.8762	1.1285	0.1977	0.109*	
O4	0.1513 (2)	0.5644 (2)	0.3745 (2)	0.0666 (5)	
H4	0.2444	0.5347	0.3643	0.100*	
N1	0.4677 (2)	0.4420 (2)	0.3533 (2)	0.0523 (5)	
N2	0.0236 (2)	0.7950 (2)	0.1324 (2)	0.0516 (5)	
H2	0.0807	0.7494	0.2034	0.062*	
N3	−0.1361 (2)	0.8523 (2)	0.1406 (2)	0.0509 (5)	
C1	0.5370 (3)	0.3362 (3)	0.4623 (3)	0.0624 (7)	
H1	0.4761	0.3085	0.5321	0.075*	
C2	0.6963 (3)	0.2631 (3)	0.4795 (3)	0.0694 (8)	
H2A	0.7393	0.1882	0.5581	0.083*	
C3	0.7866 (3)	0.3033 (3)	0.3797 (3)	0.0672 (8)	
H3A	0.8928	0.2558	0.3895	0.081*	
C4	0.7203 (3)	0.4173 (3)	0.2607 (3)	0.0528 (6)	
C5	0.8077 (3)	0.4653 (3)	0.1532 (3)	0.0638 (8)	
H5	0.9143	0.4225	0.1589	0.077*	
C6	0.7356 (3)	0.5740 (3)	0.0418 (3)	0.0646 (8)	
H6	0.7940	0.6059	−0.0282	0.078*	
C7	0.5749 (3)	0.6397 (3)	0.0292 (3)	0.0540 (6)	
H7	0.5283	0.7127	−0.0493	0.065*	
C8	0.4869 (3)	0.5969 (3)	0.1319 (2)	0.0451 (6)	
C9	0.5576 (3)	0.4842 (3)	0.2515 (2)	0.0449 (6)	
C10	0.2571 (3)	0.7563 (3)	0.0056 (2)	0.0486 (6)	
H10A	0.2909	0.7093	−0.0627	0.058*	
H10B	0.2884	0.8376	−0.0199	0.058*	
C11	0.0854 (3)	0.8127 (3)	0.0119 (2)	0.0455 (6)	
C12	−0.1908 (3)	0.8900 (3)	0.2445 (2)	0.0463 (6)	
C13	−0.0992 (3)	0.8864 (3)	0.3513 (3)	0.0587 (7)	
H13A	−0.0081	0.7983	0.3722	0.088*	
H13B	−0.1597	0.8897	0.4298	0.088*	
H13C	−0.0712	0.9689	0.3203	0.088*	
C14	−0.3614 (3)	0.9510 (3)	0.2510 (2)	0.0447 (6)	
C15	−0.4388 (3)	1.0446 (3)	0.3227 (3)	0.0577 (7)	
H15	−0.3831	1.0654	0.3729	0.069*	
C16	−0.5971 (3)	1.1082 (3)	0.3218 (3)	0.0640 (8)	
H16	−0.6464	1.1716	0.3705	0.077*	
C17	−0.6820 (3)	1.0784 (3)	0.2495 (2)	0.0508 (6)	
C18	−0.6079 (3)	0.9839 (3)	0.1787 (3)	0.0578 (7)	
H18	−0.6644	0.9622	0.1300	0.069*	
C19	−0.4494 (3)	0.9211 (3)	0.1796 (3)	0.0554 (7)	
H19	−0.4006	0.8573	0.1313	0.067*	
C20	0.0957 (4)	0.4729 (4)	0.3498 (5)	0.1075 (14)	
H20A	0.1292	0.3811	0.4202	0.161*	
H20B	0.1338	0.4576	0.2664	0.161*	
H20C	−0.0144	0.5168	0.3456	0.161*	

### Atomic displacement parameters (Å^2^)

**Table:**

	*U*^11^	*U*^22^	*U*^33^	*U*^12^	*U*^13^	*U*^23^
O1	0.0323 (9)	0.0558 (10)	0.0498 (10)	−0.0091 (7)	−0.0026 (7)	−0.0074 (8)
O2	0.0498 (11)	0.0737 (13)	0.0533 (11)	−0.0199 (9)	−0.0132 (9)	−0.0100 (9)
O3	0.0358 (10)	0.0972 (16)	0.0839 (15)	−0.0128 (10)	−0.0033 (9)	−0.0429 (12)
O4	0.0499 (11)	0.0642 (12)	0.0743 (13)	−0.0161 (10)	0.0034 (10)	−0.0174 (10)
N1	0.0471 (12)	0.0523 (12)	0.0533 (12)	−0.0163 (10)	−0.0040 (10)	−0.0142 (10)
N2	0.0341 (11)	0.0552 (13)	0.0525 (13)	−0.0051 (9)	−0.0079 (9)	−0.0144 (10)
N3	0.0346 (11)	0.0521 (12)	0.0560 (13)	−0.0064 (9)	−0.0049 (9)	−0.0166 (10)
C1	0.0664 (18)	0.0619 (17)	0.0547 (16)	−0.0257 (15)	−0.0087 (13)	−0.0096 (14)
C2	0.0640 (19)	0.0627 (18)	0.0703 (19)	−0.0161 (15)	−0.0258 (16)	−0.0099 (15)
C3	0.0456 (16)	0.0624 (18)	0.085 (2)	−0.0084 (13)	−0.0224 (15)	−0.0211 (16)
C4	0.0409 (14)	0.0533 (15)	0.0679 (17)	−0.0133 (12)	−0.0092 (12)	−0.0268 (13)
C5	0.0331 (13)	0.0740 (19)	0.086 (2)	−0.0173 (13)	0.0023 (14)	−0.0330 (17)
C6	0.0447 (15)	0.0720 (19)	0.077 (2)	−0.0245 (14)	0.0125 (14)	−0.0252 (16)
C7	0.0457 (14)	0.0550 (15)	0.0571 (16)	−0.0182 (12)	0.0020 (12)	−0.0152 (12)
C8	0.0355 (12)	0.0448 (13)	0.0546 (14)	−0.0122 (10)	−0.0009 (11)	−0.0194 (11)
C9	0.0383 (13)	0.0446 (13)	0.0544 (15)	−0.0132 (11)	−0.0038 (11)	−0.0211 (11)
C10	0.0424 (13)	0.0505 (14)	0.0477 (14)	−0.0162 (11)	−0.0022 (11)	−0.0114 (11)
C11	0.0408 (13)	0.0409 (13)	0.0514 (14)	−0.0118 (10)	−0.0062 (11)	−0.0134 (11)
C12	0.0388 (13)	0.0423 (13)	0.0495 (14)	−0.0100 (10)	−0.0044 (11)	−0.0104 (11)
C13	0.0438 (14)	0.0680 (18)	0.0578 (16)	−0.0124 (13)	−0.0077 (12)	−0.0215 (14)
C14	0.0385 (12)	0.0462 (13)	0.0431 (13)	−0.0127 (10)	−0.0031 (10)	−0.0103 (11)
C15	0.0410 (14)	0.0717 (18)	0.0655 (17)	−0.0133 (13)	−0.0071 (12)	−0.0362 (15)
C16	0.0456 (15)	0.0753 (19)	0.0713 (18)	−0.0074 (13)	−0.0030 (13)	−0.0427 (16)
C17	0.0358 (13)	0.0590 (16)	0.0503 (14)	−0.0131 (11)	−0.0026 (11)	−0.0147 (12)
C18	0.0463 (15)	0.0756 (19)	0.0649 (17)	−0.0263 (14)	−0.0011 (12)	−0.0349 (15)
C19	0.0450 (15)	0.0657 (17)	0.0630 (16)	−0.0187 (13)	0.0031 (12)	−0.0341 (14)
C20	0.088 (3)	0.082 (3)	0.168 (4)	−0.045 (2)	0.025 (3)	−0.052 (3)

### Geometric parameters (Å, °)

**Table:**

O1—C8	1.371 (3)	C7—C8	1.364 (3)
O1—C10	1.420 (3)	C7—H7	0.9300
O2—C11	1.229 (3)	C8—C9	1.423 (3)
O3—C17	1.361 (3)	C10—C11	1.501 (3)
O3—H3	0.8200	C10—H10A	0.9700
O4—C20	1.389 (4)	C10—H10B	0.9700
O4—H4	0.8200	C12—C14	1.490 (3)
N1—C1	1.319 (3)	C12—C13	1.501 (3)
N1—C9	1.369 (3)	C13—H13A	0.9600
N2—C11	1.333 (3)	C13—H13B	0.9600
N2—N3	1.396 (3)	C13—H13C	0.9600
N2—H2	0.8600	C14—C15	1.379 (3)
N3—C12	1.286 (3)	C14—C19	1.387 (3)
C1—C2	1.399 (4)	C15—C16	1.380 (4)
C1—H1	0.9300	C15—H15	0.9300
C2—C3	1.352 (4)	C16—C17	1.372 (4)
C2—H2A	0.9300	C16—H16	0.9300
C3—C4	1.413 (4)	C17—C18	1.372 (4)
C3—H3A	0.9300	C18—C19	1.382 (4)
C4—C5	1.410 (4)	C18—H18	0.9300
C4—C9	1.420 (3)	C19—H19	0.9300
C5—C6	1.353 (4)	C20—H20A	0.9600
C5—H5	0.9300	C20—H20B	0.9600
C6—C7	1.404 (4)	C20—H20C	0.9600
C6—H6	0.9300		
			
C8—O1—C10	116.27 (18)	C11—C10—H10B	109.1
C17—O3—H3	109.5	H10A—C10—H10B	107.9
C20—O4—H4	109.5	O2—C11—N2	124.5 (2)
C1—N1—C9	117.8 (2)	O2—C11—C10	117.8 (2)
C11—N2—N3	118.39 (19)	N2—C11—C10	117.4 (2)
C11—N2—H2	120.8	N3—C12—C14	115.1 (2)
N3—N2—H2	120.8	N3—C12—C13	125.8 (2)
C12—N3—N2	116.1 (2)	C14—C12—C13	118.9 (2)
N1—C1—C2	124.1 (3)	C12—C13—H13A	109.5
N1—C1—H1	117.9	C12—C13—H13B	109.5
C2—C1—H1	117.9	H13A—C13—H13B	109.5
C3—C2—C1	118.7 (3)	C12—C13—H13C	109.5
C3—C2—H2A	120.6	H13A—C13—H13C	109.5
C1—C2—H2A	120.6	H13B—C13—H13C	109.5
C2—C3—C4	120.3 (3)	C15—C14—C19	117.1 (2)
C2—C3—H3A	119.9	C15—C14—C12	121.7 (2)
C4—C3—H3A	119.9	C19—C14—C12	121.1 (2)
C5—C4—C3	123.2 (2)	C14—C15—C16	121.5 (2)
C5—C4—C9	119.7 (2)	C14—C15—H15	119.2
C3—C4—C9	117.1 (3)	C16—C15—H15	119.2
C6—C5—C4	119.7 (2)	C17—C16—C15	120.4 (2)
C6—C5—H5	120.2	C17—C16—H16	119.8
C4—C5—H5	120.2	C15—C16—H16	119.8
C5—C6—C7	121.7 (3)	O3—C17—C18	122.9 (2)
C5—C6—H6	119.2	O3—C17—C16	117.8 (2)
C7—C6—H6	119.2	C18—C17—C16	119.3 (2)
C8—C7—C6	120.2 (3)	C17—C18—C19	120.0 (2)
C8—C7—H7	119.9	C17—C18—H18	120.0
C6—C7—H7	119.9	C19—C18—H18	120.0
C7—C8—O1	124.3 (2)	C18—C19—C14	121.6 (2)
C7—C8—C9	120.1 (2)	C18—C19—H19	119.2
O1—C8—C9	115.5 (2)	C14—C19—H19	119.2
N1—C9—C4	122.0 (2)	O4—C20—H20A	109.5
N1—C9—C8	119.4 (2)	O4—C20—H20B	109.5
C4—C9—C8	118.6 (2)	H20A—C20—H20B	109.5
O1—C10—C11	112.4 (2)	O4—C20—H20C	109.5
O1—C10—H10A	109.1	H20A—C20—H20C	109.5
C11—C10—H10A	109.1	H20B—C20—H20C	109.5
O1—C10—H10B	109.1		
			
C11—N2—N3—C12	−151.2 (2)	O1—C8—C9—C4	179.2 (2)
C9—N1—C1—C2	−1.0 (4)	C8—O1—C10—C11	179.3 (2)
N1—C1—C2—C3	0.9 (5)	N3—N2—C11—O2	2.2 (4)
C1—C2—C3—C4	0.0 (5)	N3—N2—C11—C10	177.3 (2)
C2—C3—C4—C5	179.6 (3)	O1—C10—C11—O2	−167.4 (2)
C2—C3—C4—C9	−0.7 (4)	O1—C10—C11—N2	17.2 (3)
C3—C4—C5—C6	179.2 (3)	N2—N3—C12—C14	179.9 (2)
C9—C4—C5—C6	−0.5 (4)	N2—N3—C12—C13	4.1 (4)
C4—C5—C6—C7	−0.8 (4)	N3—C12—C14—C15	−154.8 (3)
C5—C6—C7—C8	1.5 (4)	C13—C12—C14—C15	21.3 (4)
C6—C7—C8—O1	179.5 (2)	N3—C12—C14—C19	22.1 (3)
C6—C7—C8—C9	−0.7 (4)	C13—C12—C14—C19	−161.8 (2)
C10—O1—C8—C7	6.5 (3)	C19—C14—C15—C16	−1.2 (4)
C10—O1—C8—C9	−173.2 (2)	C12—C14—C15—C16	175.9 (3)
C1—N1—C9—C4	0.2 (4)	C14—C15—C16—C17	0.5 (5)
C1—N1—C9—C8	179.3 (2)	C15—C16—C17—O3	179.8 (3)
C5—C4—C9—N1	−179.7 (2)	C15—C16—C17—C18	0.4 (4)
C3—C4—C9—N1	0.6 (4)	O3—C17—C18—C19	180.0 (3)
C5—C4—C9—C8	1.2 (4)	C16—C17—C18—C19	−0.7 (4)
C3—C4—C9—C8	−178.5 (2)	C17—C18—C19—C14	0.0 (4)
C7—C8—C9—N1	−179.7 (2)	C15—C14—C19—C18	0.9 (4)
O1—C8—C9—N1	0.1 (3)	C12—C14—C19—C18	−176.1 (2)
C7—C8—C9—C4	−0.6 (4)		

### Hydrogen-bond geometry (Å, °)

**Table:**

*D*—H···*A*	*D*—H	H···*A*	*D*···*A*	*D*—H···*A*
O3—H3···O2^i^	0.82	1.85	2.647 (3)	165
O4—H4···N1	0.82	1.96	2.773 (3)	174
O4—H4···O1	0.82	2.60	3.036 (3)	115
N2—H2···O4	0.86	2.10	2.856 (3)	146

Symmetry codes: (i) −*x*−1, −*y*+2, −*z*.
